# The Alberta moving beyond breast cancer (AMBER) cohort study: a prospective study of physical activity and health-related fitness in breast cancer survivors

**DOI:** 10.1186/1471-2407-12-525

**Published:** 2012-11-16

**Authors:** Kerry S Courneya, Jeff K Vallance, S Nicole Culos-Reed, Margaret L McNeely, Gordon J Bell, John R Mackey, Yutaka Yasui, Yan Yuan, Charles E Matthews, David CW Lau, Diane Cook, Christine M Friedenreich

**Affiliations:** 1 Faculty of Physical Education and Recreation, University of Alberta, Edmonton, Canada; 2 Faculty of Health Disciplines, Athabasca University, Athabasca, Canada; 3 Faculty of Kinesiology, University of Calgary, Calgary, Canada; 4 Faculty of Rehabilitation Medicine, University of Alberta, Edmonton, Canada; 5 Faculty of Medicine and Dentistry, University of Alberta, Edmonton, Canada; 6 School of Public Health, University of Alberta, Edmonton, Canada; 7 Division of Cancer Epidemiology and Genetics, US National Cancer Institute, Bethesda, Maryland; 8 Faculty of Medicine, University of Calgary, Calgary, Canada; 9 Department of Epidemiology, Alberta Health Services, Calgary, Canada; 10 Faculty of Physical Education and Recreation, University of Alberta, E-488 Van Vliet Center, Edmonton, AB, Canada

**Keywords:** Breast cancer, Exercise, Physical activity, Cardiorespiratory fitness, Muscular strength, Lymphedema, Quality of life, Exercise determinants, Recurrence, Survival

## Abstract

**Background:**

Limited research has examined the association between physical activity, health-related fitness, and disease outcomes in breast cancer survivors. Here, we present the rationale and design of the Alberta Moving Beyond Breast Cancer (AMBER) Study, a prospective cohort study designed specifically to examine the role of physical activity and health-related fitness in breast cancer survivorship from the time of diagnosis and for the balance of life. The AMBER Study will examine the role of physical activity and health-related fitness in facilitating treatment completion, alleviating treatment side effects, hastening recovery after treatments, improving long term quality of life, and reducing the risks of disease recurrence, other chronic diseases, and premature death.

**Methods/Design:**

The AMBER Study will enroll 1500 newly diagnosed, incident, stage I-IIIc breast cancer survivors in Alberta, Canada over a 5 year period. Assessments will be made at baseline (within 90 days of surgery), 1 year, and 3 years consisting of objective and self-reported measurements of physical activity, health-related fitness, blood collection, lymphedema, patient-reported outcomes, and determinants of physical activity. A final assessment at 5 years will measure patient-reported data only. The cohort members will be followed for an additional 5 years for disease outcomes.

**Discussion:**

The AMBER cohort will answer key questions related to physical activity and health-related fitness in breast cancer survivors including: (1) the independent and interactive associations of physical activity and health-related fitness with disease outcomes (e.g., recurrence, breast cancer-specific mortality, overall survival), treatment completion rates, symptoms and side effects (e.g., pain, lymphedema, fatigue, neuropathy), quality of life, and psychosocial functioning (e.g., anxiety, depression, self-esteem, happiness), (2) the determinants of physical activity and health-related fitness including demographic, medical, social cognitive, and environmental variables, (3) the mediators of any observed associations between physical activity, health-related fitness, and health outcomes including biological, functional, and psychosocial, and (4) the moderators of any observed associations including demographic, medical, and biological/disease factors. Taken together, these data will provide a comprehensive inquiry into the outcomes, determinants, mechanisms, and moderators of physical activity and health-related fitness in breast cancer survivors.

## Background

Breast cancer is a major public health burden in Canada with 23,600 women expected to be diagnosed in 2011 and 5,100 expected to die from the disease [1]. Over their lifetime, Canadian women have about a one-in-nine chance of developing breast cancer and a 1 in 29 chance of dying from the disease [1]. Breast cancer accounts for 28% of all cancers diagnosed in women and 14% of all cancer deaths in women [1]. Early detection and improved treatments have resulted in a five-year relative survival rate of 88% [1]. The high incidence rate and excellent survival rate have resulted in a growing population of breast cancer survivors. In 2007, there were an estimated 153,000 breast cancer survivors in Canada diagnosed within the past 10 years, comprising 40% of all female cancer survivors in Canada [1]. Given only survivors up to 10 years post-diagnosis were included in the estimate, it is likely that there are over 200,000 breast cancer survivors currently in Canada. Breast cancer survivors are at increased risk for many acute, chronic, and late effects of their disease and treatments including breast cancer recurrence, second cancers, cardiac dysfunction, weight gain, bone loss, lymphedema, arthralgias, cognitive dysfunction, menopausal symptoms, fatigue, and psychosocial distress [2].

Physical activity (PA) and health-related fitness (HRF) are essential for the health of any population but they appear to be particularly important for breast cancer survivors. A growing body of literature has examined the associations between PA and disease outcomes in breast cancer survivors and the preliminary results are promising [3]. These studies are limited, however, because few were originally designed as breast cancer survivor cohorts and none were designed with a primary focus on PA and HRF [3]. Consequently, these cohort studies have methodological limitations including a reliance on self-report PA measures that do not assess lifetime PA and other important domains of PA (e.g., occupational), no objective assessments of PA or HRF, no measure of sedentary behavior, limited investigation of potential biomarkers, and unstandardized assessment time points.

Here, we report the design and methods of the Alberta Moving Beyond Breast Cancer (AMBER) Study. To the best of our knowledge, the AMBER Study is the first prospective cohort study designed specifically to examine the role of PA and HRF in breast cancer survivorship. The AMBER Study will address several gaps in the literature by including:

1) a comprehensive self-report measure of PA [4,5] that assesses the type, frequency, intensity, and duration of PA at work, at home, and for recreation and transportation;

2) a self-report assessment of sedentary behavior, which is emerging as an important independent predictor of disease outcomes including cancer [6];

3) objectively-determined PA and sedentary behavior (i.e., accelerometers);

4) a comprehensive assessment of HRF components including standardized and validated measures of maximal cardiorespiratory fitness, musculoskeletal fitness, and body composition;

5) a comprehensive assessment of upper extremity range of motion, testing of sensorimotor function including balance, and surveillance of arm volume and symptoms for the early detection of lymphedema,

6) an assessment of biomarkers purported to mediate the possible associations between PA, HRF, and breast cancer outcomes;

7) assessment of key PROs including quality of life, fatigue, and psychosocial function using standardized and validated measures; and

8) a theoretical model of human behavior to examine the determinants of PA.

Consequently, the AMBER Study will provide the most comprehensive inquiry into the role of PA and HRF in breast cancer survivorship to date. In the following sections, we describe the methods and design of the AMBER Study and discuss the five main projects that guided its initial development.

## Methods

### Study design

**Figure 1:**
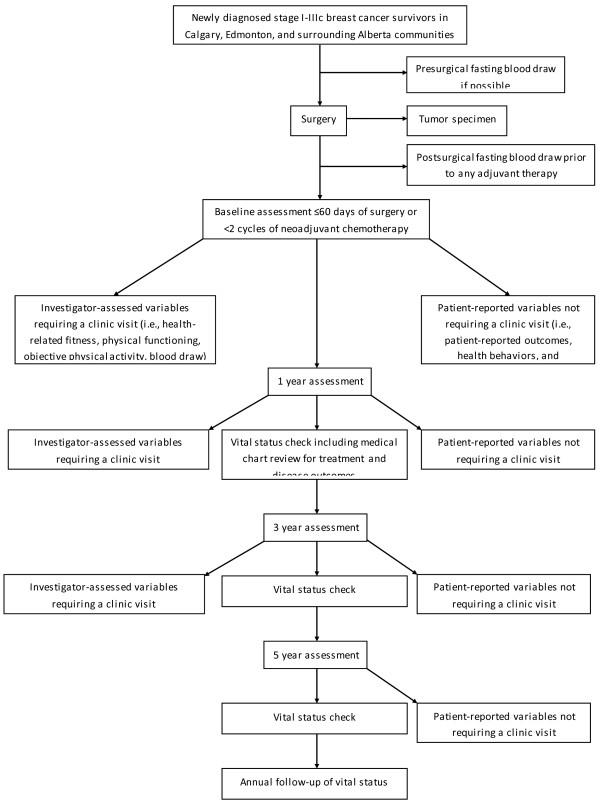
*Flow of participants through the AMBER cohort study.*

The AMBER study protocol was approved by the Alberta Cancer Research Ethics Committee and all participants are required to provide written informed consent. The AMBER Study is a prospective cohort study of newly diagnosed breast cancer survivors in Alberta, Canada. Flow through the study is depicted in Figure 1. Assessments will be made at baseline, and one, three, and five years follow-up and include clinic-based and patient-reported measures. The clinic-based measures will be completed at baseline, one and three years whereas the patient-reported measures will be completed at all time points. The baseline assessment will be completed within three months of definitive breast cancer surgery. The baseline blood draw will be pre-surgical whenever possible, however, a post-surgical blood draw will be acceptable as long as it is prior to the start of any adjuvant therapy. For all other baseline assessments, the goal is to complete them prior to any adjuvant therapy, however, women may complete them after the start of adjuvant therapy as long as they are still within three months of surgery and have received less than one chemotherapy cycle or less than two weeks of radiation therapy. For women receiving neoadjuvant chemotherapy, the baseline blood sample will be drawn prior to the start of chemotherapy whereas all other baseline measures will be completed before the second cycle of chemotherapy. The one-year assessment is intended to capture the short-term effects of treatments on the various health outcomes. This approach was selected over a post-treatment time point given the highly varied length of breast cancer treatments (i.e., from months to years). The three-year assessment is intended to capture the early survivorship recovery period and reflect a more stable estimate of the various measures. The five-year assessment will provide a longer term follow-up of self-reported PA, PA determinants, and patient-reported outcomes. After five years, study participants will be followed passively for vital status including progressions, recurrences, and new primaries through regular linkages with vital status data maintained by the Alberta Cancer Registry (ACR).

### Study population

Eligibility for the AMBER Study includes: (1) histologically-confirmed stage 1 (T1cN0M0) to stage IIIc breast cancer [7], (2) no prior cancer diagnosis except non-melanoma skin cancer, (3) females ≥ 18 years old, (4) completing the revised Physical Activity Readiness Questionnaire for Everyone (rPAR-Q+) [8] and the electronic Physical Activity Readiness Medical Examination questionnaire (ePARmed-X+) [8]; in order to complete health-related fitness testing, (5) living in and around two large metropolitan centres, Edmonton and Calgary (and surrounding areas), (6) ability to provide written informed consent and complete questionnaires in English, and (7) not pregnant. We will include stage 1 (T1cN0M0) to stage IIIc breast cancer survivors to ensure that we achieve a broad sample of the breast cancer survivorship community while ensuring an adequate number of recurrences in this cohort.

### Recruitment

Breast cancer survivors in Alberta being evaluated at the Cross Cancer Institute in Edmonton and the Tom Baker Cancer Centre in Calgary will be recruited. These two cities include two-thirds of all breast cancer cases in Alberta. Alberta Health Services has developed a rapid breast cancer ascertainment method in Calgary, in conjunction with the Alberta Cancer Research Biorepository (ACRB) that uses a population-based sampling approach to identify all breast cancer cases prior to surgery. In Calgary, all newly diagnosed women are contacted by the ACRB to provide a pre-surgical blood and tumour tissue sample. If consent is obtained, their blood and tissue samples are stored for future research purposes. A letter of invitation, information brochure, and consent form will be e-mailed to the potential study participants and a telephone follow-up is used to recruit into the study. Those that are interested in the study are administered the rPAR-Q + by telephone by our certified exercise physiologists prior to fitness testing. In Edmonton, breast cancer survivors are recruited from the New Patient Breast Clinic at the Cross Cancer Institute. Prospective participants are provided with a letter of invitation, information brochure, consent form and rPAR-Q + and asked to return it when they attend the clinic for testing.

### Measures

Appointments are made with Edmonton participants to come to the Cross Cancer Institute and Behavioral Medicine Fitness Center at the University of Alberta, and with Calgary participants to come to the Tom Baker Cancer Center and the Human Performance Laboratory at Faculty of Kinesiology at the University of Calgary. The assessments will be scheduled for one or two clinic visits depending on participant preference and logistical issues. Single day clinic visits will be split into morning and afternoon assessments to avoid undue fatigue. The assessments will include 1) questionnaires, (e.g., baseline demographic and health history as well as patient-reported outcomes), (2) review of the rPAR-Q + and completion of the ePARmed-X + form, (3) completion of HRF testing including DXA scans, (4) lymphedema/upper body function measurements, (5) blood draw, and (6) training in the use of the accelerometer and activity monitor logs to complete a week-long objective PA measurements.

### Health and lifestyle questionnaires

#### Baseline health

Baseline demographic and health characteristics of the study participants will be measured by self-administered questionnaire and will include demographic characteristics (marital status, education, income, employment status, ethnicity), menopausal status, menstrual and reproductive history, exogenous hormone use history, personal health history, medication history, vitamin and supplement history, family history of cancer, and smoking and alcohol drinking histories.

#### Physical activity

Self-reported PA will be assessed by the self-administered *Past Year Total Physical Activity Questionnaire*[5]. This questionnaire has been used in over 30,000 participants in the Alberta Cohort Study (Tomorrow Project®) [9] and measures all types and parameters of activity over the past 12 months. For this questionnaire, the recorded activity is converted into MET-hours/week/year of activity performed using the Compendium of Physical Activities developed by Ainsworth and colleagues [10].

#### Sedentary behavior

Self-reported sedentary behavior will be assessed by a questionnaire that has been adapted from the Australian Longitudinal Study on Women’s Health and has been shown to have acceptable reliability and validity [11]. The questionnaire includes five items assessing time spent sitting (hours and minutes) each day in the following domains: a) while traveling to and from places, b) while at work, c) while watching television, d) while using a computer at home, and e) at leisure not including watching television, on a weekday and a weekend day.

#### Dietary habits

Usual dietary intake in the previous 12 months will be assessed using the Canadian version of the US National Cancer Institute’s *Diet History Questionnaire*[12] that has recently been updated to reflect changes in food practices and nutrient content of foods. Due to its cognitive-based design, this questionnaire captures usual intake better than other well-tested and validated questionnaires, such as the Block and Willett food frequency questionnaires [13]. Diet is an important potential confounder given that total energy intake can affect energy balance (i.e., body composition) along with PA. Furthermore, dietary composition could affect markers of inflammation and insulin resistance [14], two biomechanisms of interest in our study, and hence will be controlled for in the analysis.

### Patient-reported outcomes

*Health-Related Quality of life (HRQoL)* will be assessed by the SF-36 version 2 which is a widely used self-report measure designed to assess perceived health and functioning [15,16]. It contains eight subscales labeled Physical Functioning, Role-Physical; Bodily Pain; General Health; Vitality; Social Functioning; Mental Health; and Role-Emotional. A physical component summary and a mental component summary are also generated. Scales are comprised of different numbers of items and use a variety of rating formats. Raw scores are converted to a standard metric (0–100), with higher scores being indicative of a better health state. The validity and reliability of the SF-36 has been established in a number of clinical populations, including cancer patients [17]. The standard four-week version of the SF-36 will be used.

*Fatigue* will be assessed using the Fatigue Scale (FS) [18] from the FACT measurement system. The FS contains 13 items that measure the impact of fatigue on cancer survivors. On the FS, higher scores represent less fatigue, or less severe symptoms. All FS items are rated on a 5-point Likert scale ranging from 0 (not at all) to 4 (very much). The FS assessment is brief, easy to administer, and has suitable evidence of internal consistency, test-retest reliability, and convergent and discriminant validity, and clinically important thresholds have been derived [18,19].

*Depression symptoms* will be assessed using the Patient Health Questionnaire – 9 (PHQ-9) [20]. The PHQ-9 scores each of the 9 DSM-IV criteria and ranges from “0” (not at all) to “3” (nearly every day). Items are preceded by the statement “Over the last 2 weeks, how often have you been bothered by any of the following problems?” [20]*Satisfaction with life* will be assessed using Diener’s Satisfaction With Life Scale (SWL) [21]. The SWLS is a short 5-item instrument designed to measure global cognitive judgments of satisfaction with one's life. *Happiness* will be measured using the Happiness Measure (HM) [22]. The HM contains a question asking for the amount of time spent happy, unhappy, and neutral in the past week. Percentage of time spent happy in the previous week will be estimated.

### Determinants of physical activity

The social cognitive determinants of PA behavior will be assessed by self-administered questionnaires using the Theory of Planned Behavior variables of *attitude* (affective and instrumental), *subjective norm* (injunctive and descriptive), *perceived behavioral control* (self-efficacy and perceived control), *intentions*, and *planning*. These measurements are in accordance with Ajzen’s suggestions [23] and have been shown to be reliable and valid within cancer populations [24-26]. In addition, barriers to exercise will be assessed, including disease/treatment-related, life-related and motivation-related barriers. Environmental determinants of PA behavior will include examination of the relationship between location (postal code), neighborhood walkability, and access to fitness facilities.

### Objective measurements of physical activity and sedentary behavior

PA and sedentary time will be measured objectively in all study participants using the new Actigraph GT3X + (Actigraph, LLC, Pensacola, FL). This small and lightweight device is a highly sensitive instrument that records acceleration using a tri-axial accelerometer. These data are digitized by a 12-bit A/D converter 30 times per second (30 Hz), filtered to capture normal human movement (i.e., 0.25 to 2.5 Hz), and saved as an activity count in user defined intervals (epochs). Activity counts (ct) provide an indication of the duration and intensity of bodily movement. These data will be summarized using established data reduction methods [27,28], artificial neural network algorithms that predict activity type (e.g. household, locomotion, sport) and intensity (metabolic equivalents [METs]) [29], as well as traditional scoring methods to estimate activity duration (hrs/d) using activity count thresholds (i.e., sedentary, light, moderate-vigorous). Participants will be instructed to wear the monitor on their right hip for seven consecutive days during all waking hours, except while bathing or swimming. Additionally, they will be asked to record, in a daily log, the time they put on and took off the monitor each day and the activities they did when they were not wearing a monitor. The participants will be instructed in the use of the accelerometers after their HRF assessments and will return the monitor and the logbook to the study coordinator. Study participants will be asked to wear the accelerometers at baseline, 1, and 3 years.

### Health-related fitness assessments

The HRF assessments will be performed by Canadian Society for Exercise Physiology Certified Exercise Physiologists (CSEP:CEP®) using the same testing equipment in both centres and following a detailed testing protocol. They will complete the assessments in the following order during a morning session: resting BP and HR; body composition/anthropometry; [using dual x-ray absorptiometry (DXA)]; musculoskeletal fitness and cardio-respiratory fitness. Specific upper and lower body (chest and leg press) muscular strength and endurance will be tested in the afternoon after adequate recovery if the participant chooses single day testing. Otherwise the muscular strength and endurance testing may be performed the following visit. Adequate recovery time and nutrition/ hydration will be provided to ensure that accumulated fatigue is minimized. On the morning of the first visit, the DXA scan and lymphedema assessments will be performed. The total HRF assessment time will be approximately 2–2.5 hours per day for two day testing and 6–6.5 hours total over the morning and afternoon for single day testing.

#### Resting heart rate and blood pressure

Using standardized procedures [30], after 5 minutes of quiet sitting, resting heart rate will be measured using a heart rate monitor (Polar Electro, Finland) and resting blood pressure will be measured on the non-surgical arm using a sphygmomanometer (WelchAllyn®) and stethoscope (3M^TM^ Littmann®).

#### Body composition/athropometry

Standing height (stadiometer, North Bend, WA) and body mass (HealthoMeter, Bradford, MA) without shoes will be measured. Waist circumference will be measured using the National Institutes of Health (NIH) protocol and hip circumference will be measured using the World Health Organization (WHO) procedure with an anthropometric tape measure (Gulick tape measure, Gilroy, CA). Waist to hip ratio and body mass index (kg × m^-2^) will be calculated [30]. A DXA scan with a full body image will be taken to assess overall percent body fat, total lean body mass, total fat mass, and bone mineral density.

#### Cardiorespiratory fitness

Submaximal heart rate, blood pressure, blood oxygen saturation (SpO_2_, Vacumed, Venura CA), rating of perceived exertion (Borg CR10 scale) [31], ventilatory threshold (VT) and peak oxygen uptake (VO_2_ peak) will be determined during a modified Bruce [32] graded exercise test on a treadmill. The modified Bruce treadmill protocol will be used because of the nature of the participants (e.g., large age range, varied fitness levels). During each treadmill test, all expired air will be collected and analyzed with a calibrated TrueOne metabolic measurement system (ParVo Medics Inc, Sandy, UT.). VT will be determined using the V-slope method of Wasserman [33] and VO_2_ peak will be determined as the highest oxygen uptake achieved during the treadmill test to volitional exhaustion. Recovery heart rate and systolic blood pressure will be measured 1 and 5 minutes after the treadmill test ends.

*Muscular strength* will be assessed using multiple repetition maximum (mRM) strength tests for chest press and leg press to determine the maximum weight an individual can lift approximately 8 – 10 times. The protocol will follow the National Strength and Conditioning Association guidelines [34]. The mRM will be used to predict a 1RM using the formulae reported by Mayhew et al. [35]. Combined grip strength (kg) of the right and left hands will be measured using a JAMAR hydraulic hand dynamometer (Lafayette Instruments, ID). A sum will also be calculated from the best score of 2 trials recorded for each hand according to the CPAFLA protocol [30].

*Muscular Endurance* of the abdominals will be assessed by a partial curl-up test and will be performed according to a standardized protocol [30,36]. Upper body muscular endurance will be assessed using the chest press exercise device described previously at a relative load of 50% of predicted 1RM. Lower body muscular endurance will be done using leg press at 70% of predicted 1RM.

*Flexibility* will be assessed by a trunk forward flexion sit-and-reach test using a Wells-Dillon flexometer according to a standardized protocol [30].

### Objective measurements of physical functioning

*Lymphedema* will be assessed both by self-report and by clinical examination. Complications such as infection and venous thrombophlebitis in the limb will be recorded at the time of assessment. *Arm volume* will be measured objectively using the Perometer (Juzo, Cuyahoga Falls, USA). The Perometer is an optoelectric limb volumeter that uses infrared technology to quantify limb volume. It is a validated, reliable and sensitive method for quantifying limb volume [37,38]. This instrument provides assessments of the entire limb volume, the percentage difference between limbs, and allows for inter-limb comparison over time.

#### Range of motion

The measurement of *shoulder range of motion* will be performed by using a universal goniometer according to standardized procedures [39]. Active and passive shoulder movements will be measured and include the measurements of forward flexion, abduction, internal rotation, external rotation and horizontal abduction movements.

#### Arm function

*Arm function* will be assessed using the Disabilities of the Arm, Shoulder and Hand scale (DASH) [40]. The scale measures the effect of arm function on 30 different daily activities. It also examines symptoms such as pain, weakness and numbness, and the degree of disability related to work and recreational activity. The scale is scored from 0 to 100 with higher scores indicating greater disability. This scale has been well documented and shown to be reliable and valid.

#### Peripheral neuropathy

Peripheral neuropathy will be assessed by self-report and objective measures of sensorimotor function, strength and balance testing.

### Blood data collection

The baseline blood draw will be done after an overnight fast of at least eight hours. A 60 ml sample will be taken at baseline and a 30 ml sample will be taken at 1 and 3 year follow-ups. For this study, we will store 24 aliquots per person for baseline blood draw (14 serum, 6 plasma, 2 buffy coat and 2 red blood cells) and 16 aliquots per person for 1 and 3 year follow-up blood draw (8 serum, 5 plasma, 1 buffy coat and 2 red blood cells). A complete blood collection, processing, shipping and storage protocol has been developed to ensure standardization of the procedures for the bloods at the collection sites in Calgary (Calgary Laboratory Services) and Edmonton (Cross Cancer Institute). The aliquoted blood samples will be stored in our −86°C freezers.

### Medical record abstraction

Health Record Technicians from the Alberta Cancer Registry will use standardized forms and methods to abstract the medical charts for all of the participants at regular intervals during the cohort study. The form was previously developed and tested for our past PA and breast cancer cohort study that evolved from a population-based case–control study that we conducted in Alberta [41]. Medical variables to be abstracted will include pathologic and clinical disease stage (TNM), type of surgery, and all treatment and follow-up care including data on chemotherapy, radiation therapy, and hormone therapy. Pathology data will include tumor size, grade, histology, estrogen receptor status, human epidermal growth factor receptor 2 (HER2-neu) status, type and results of computerized tomography (CT) or positron emission tomography (PET) scans, status of margins (with breast conserving surgery), and pathology of lymph nodes (if surgically sampled).

*Treatment completion rates* will be estimated for chemotherapy and hormone therapy but not for radiation therapy since few survivors fail to complete radiation therapy. For chemotherapy completion rate, we will estimate the average relative dose intensity (RDI) received for the originally planned regimen based on standard formulae as we have done in a previous RCT [42]. For hormone therapy completion rate, it is not feasible to obtain an objective measure of treatment adherence. Consequently, participants will be asked to report if they have stopped taking their prescribed hormone therapy at any time before its intended completion and the reasons for stopping their treatments.

*Disease endpoints* will be defined and assessed based on the Standardized Definitions for Efficacy End Points in Adjuvant Breast Cancer Trials (the STEEP system) [43]. We selected recurrence-free interval (RFI) as our primary disease endpoint because it consists of events directly attributed to breast cancer including invasive ipsilateral breast tumor recurrence, local/regional invasive recurrence, distant recurrence, and death from breast cancer. We will also examine other composite disease endpoints as secondary endpoints including overall survival, invasive disease-free survival, distant disease-free survival, distant relapse-free survival, breast cancer-free interval, and distant recurrence-free interval. Finally, we will examine the single disease endpoints of death from breast cancer and death from non-breast cancer. These endpoints will be abstracted by the health records technicians at the time of the medical chart reviews described above.

Information on all deaths that occurred in the province is provided by Vital Statistics Alberta (VSA) to the ACR on a monthly basis with underlying cause of death provided by Statistics Canada to VSA. There is an average three-month time lag between the actual death occurrence and reporting to the ACR. For cases that leave the province after diagnosis, several mechanisms exist to capture their deaths with reciprocal agreements between other provinces and record linkages with the Canadian Mortality Database that are undertaken with the ACR data. These agreements and processes ensure that vital status can be determined for over 95% of cases. For cases who have left the province and who are not known to be dead, the date of leaving Alberta will be used as the censoring time. For the follow-up of this cohort, yearly checks of the vital status of the breast cancer survivors will be conducted through linkages with the ACR. Cause of death and date of death will be obtained from this source.

### Sample size and power considerations

Although a cohort study such as this one has many exposures, time points, and endpoints of interest, we chose to power our study using cardiorespiratory fitness at one-year follow-up as the primary exposure/time point, and recurrence free interval (RFI) as the primary endpoint because of the importance of these variables but also because of the substantial power needed to detect this association. By designing the study with adequate power for this association, we ensured sufficient power for all the other objectives that require substantially less power. We designed the study to detect a 30% risk reduction of RFI in survivors who are in the top quartile of cardiorespiratory fitness, compared to those who are in the lowest quartile (hazard ratio = 0.7). The sample size was determined under the framework of Cox proportional hazards (PH) models, adjusting for known potential confounders such as age, BMI, tumor stage, treatment, alcohol intake, diet, smoking and co-morbidity. A statistical power of 80% and a two-sided α = 0.05 were used. This setting requires 223 to 401 events (PASS 2005); the exact number depends on how strong the association is between cardiorespiratory fitness and the confounders in the Cox PH model. A stronger association requires a larger number of events. The association among variables is quantified by *R*^*2*^ of a multiple linear regression model where cardiorespiratory fitness is regressed onto the confounders. In the above calculation, we have considered a range of *R*^*2*^ between 0.1 (requiring 223 events) and 0.5 (requiring 401 events) that we deemed realistic. The *R*^*2*^ can be reduced by using cardiorespiratory fitness quartiles that are adjusted for some of the major confounders such as age and BMI, ensuring the *R*^*2*^ value of less or equal to 0.5. Thus, our study is sufficiently powered for the primary objective if we have more than 401 events.

The annual recurrence rate for localized invasive breast cancer is estimated to be 2 ~ 3% [44]. For stage I – IIIc patients combined, a first five-year recurrence rate of 20% has been reported [45,46]. To attain the desired number of events, we are using a stratified sampling design based on the projected breast cancer cases diagnosed in 2012–15 who will be treated in Edmonton or Calgary. We plan to recruit a total of 1500 breast cancer survivors from the two locations. Given the projected numbers of survivors seen at two locations for each stage, and, assuming an overall 50% consent rate for participation, the recruitment will be completed within 5 years. This recruitment plan will provide a median of about seven years of follow-up by the end of this study in year 2022, and will yield a minimum of 460 events. Allowing for a 10% loss to follow-up, we expect to observe, at minimum, 414 events in this cohort.

## Discussion

The primary focus of the AMBER Study will be to identify the independent and interactive associations of PA and HRF with disease outcomes (e.g., recurrence, breast cancer-specific mortality, overall survival). Other important health outcomes will include treatment completion rates, symptoms and side effects (e.g., pain, lymphedema, fatigue, cognitive dysfunction), and PROs (e.g., QoL, anxiety, depression, self-esteem, happiness). We will also be able to examine the mediators and moderators of any observed associations between PA, HRF, and health outcomes. Finally, we will be able to identify the key determinants of PA and HRF including demographic, medical, and social cognitive variables, at various time points across the survivorship trajectory. Taken together, these data will provide a detailed understanding of the unique benefits, risks, and determinants of PA and HRF at multiple time points of survivorship so that intervention strategies can be developed to help breast cancer survivors achieve and maintain healthy levels of PA and HRF. The AMBER Study is designed initially to address the following five major research themes.

### Physical activity, health-related fitness, and disease outcomes

The primary aim of this project is to examine the associations between self-reported and objective PA (including sedentary behavior), HRF (including body composition), and disease outcomes in breast cancer survivors (including recurrence-free interval, breast cancer mortality, and overall survival). These data will provide critical information on the optimal type, volume, and pattern (i.e., how the volume is achieved over a given week) of PA that may be most strongly associated with disease outcomes in breast cancer survivors. Moreover, while previous studies have used self-report PA assessments, the use of accelerometers to measure PA will provide an accurate (and gold-standard) estimate of PA at multiple time points across the survivorship trajectory. Further, no studies have examined associations between objectively-determined PA and disease endpoints. Multivariable analyses will be able to determine any independent associations of the PA and HRF variables with disease outcomes that may identify one or more PA-related exposures of primary importance. For example, two research questions will be to determine whether: (a) cardiorespiratory fitness and muscular strength or other HRF assessments are independently associated with disease outcomes among breast cancer survivors, and (b) vigorous PA and sedentary behavior are independently associated with disease outcomes. A secondary aim is to examine potential moderators (effect modifiers) of the associations between PA, HRF, and disease outcomes. These data will provide critical information on which subgroups of survivors may benefit the most from engaging in PA and may also even identify different optimal PA prescriptions for different survivor subgroups. The ultimate goal of this project is to provide insights regarding the relative importance of various aspects of the PA prescription and the various HRF components for breast cancer outcomes that will be directly relevant for PA and sedentary behavior recommendations for breast cancer survivors.

### Physical activity, health-related fitness, and biologic mechanisms

The primary aim of this project is to examine the mechanisms that may explain any associations between self-reported and objective PA (including sedentary behavior), HRF (including body composition), and disease outcomes in breast cancer survivors (including recurrence-free interval, breast cancer mortality, and overall survival). The exact biologic mechanisms whereby PA and HRF may influence breast cancer recurrence and survival have not yet been delineated. More research has focused on the role of PA in breast cancer incidence. One hypothesized biologic model for postmenopausal breast cancer risk implicates adiposity, sex hormones, insulin resistance and chronic inflammation as mediators of PA [47]. This model is further supported by recent results from exercise intervention trials that demonstrated a direct impact of PA on sex hormones [48,49] and adiposity levels [50], which are both convincingly associated with postmenopausal breast cancer risk in the epidemiologic literature [47]. The same model and biologic rationale relating PA to postmenopausal breast cancer risk [47] can be adapted to breast cancer recurrence and survival since many of the same biomarkers have been associated with PA in breast cancer survivors, and breast cancer recurrence/survival, respectively. Adaptations to the model include the addition of breast cancer therapies and their potential influence on biomarkers [51,52] as well as the addition of insulin-like growth factor 1 (IGF-1) and IGF binding protein 3 (IGFBP-3). HRF (i.e., body composition, muscular strength, muscular endurance, cardiorespiratory fitness) can also be added to the model since body composition is influenced by PA, changes in muscle mass may affect insulin resistance, and in one recent healthy cohort study, cardiorespiratory fitness was found to decrease risk of breast cancer death through an unknown mechanism [53].

Clearly, there is a lack of consistent information relating PA and breast cancer outcomes to our hypothesized biomarkers. A better understanding of the underlying biologic pathways involved in the association between PA and breast cancer outcomes could be gained with a sufficiently powered study using more accurate measures of body fat, valid measures of PA, and careful control for patient and tumor-related moderators of the effects of PA on breast cancer outcome. Serial measurements of our proposed biomarkers over time will be a novel attribute of this study and will enable us to identify significant time points for influencing breast cancer outcome and the effect of biomarker level changes over time. This understanding will add biologic plausibility to the association between PA and breast cancer outcome, guide future epidemiologic research, identify new targets for interventions, and inform clinical recommendations for improving survival after breast cancer diagnosis.

### Physical activity, health-related fitness, and patient-reported outcomes

The primary aim of this project is to examine the associations between PA, HRF, and PROs across the breast cancer continuum. Breast cancer survivors have an elevated risk for poor QoL, anxiety, depression, fatigue, and cognitive impairment both during treatment [54] and throughout survivorship [55,56]. Some evidence suggests that women surviving cancer may continue to demonstrate poor function on various PROs for up to 10 years after their initial diagnosis [57-59]. While preventing declines in PROs after a breast cancer diagnosis is important, new research is suggesting that less decline in QoL during adjuvant therapy for breast cancer may also be associated with a reduced risk of breast cancer recurrence [60] (Sarenmalm et al., 2009).

Systematic reviews support the role of PA as a safe and effective intervention to improve HRF and selected PROs in breast cancer survivors, particularly during survivorship [61,62]. The most commonly studied PROs in PA research are fatigue, QoL, physical functioning, depression, and anxiety [62]. Systematic reviews provide evidence that PA can improve patient-reported physical functioning and anxiety during treatment, and QoL, fatigue, depression, and anxiety during survivorship. In particular, these studies suggest that particular QoL domains, especially physical well-being, functional well-being, and fatigue appear to be domains that are most likely affected by PA. Indeed, some data suggest that improvements in several PROs are dependent on changes in HRF such as cardiorespiratory fitness [63,64].

Although over 50 randomized controlled trials (RCTs) have examined the effects of PA on PROs in breast cancer survivors [62], few of these trials have had adequate power for subgroup analysis, few have examined the optimal *type* of PA (e.g., aerobic, resistance) or *intensity* of PA (e.g., light, moderate, vigorous activities), few have examined the HRF components most relevant to PROs, and few have examined the effects of PA or HRF on PROs across the continuum of breast cancer survivorship (e.g., treatment, early survivorship, later survivorship). Little is known about other relevant PROs such as cognitive function, taxane symptoms, hormonal symptoms, and psychological well-being (e.g., happiness and satisfaction with life). Further, to date there are no studies examining sedentary behavior (time spent sitting) and associations with PROs among breast cancer survivors. Information of this nature may facilitate further understanding of how PA and sedentary behavior is related to PROs during breast cancer survivorship. This project will also examine important mediators and moderators of the associations between PA, HRF, and PROs.

### Physical activity, health-related fitness, and physical functioning

This project will examine the relationship between PA, HRF and the incidence, severity and natural progression of lymphedema and upper limb morbidity (e.g., pain, numbness, weakness and shoulder dysfunction) from diagnosis through treatment and recovery from breast cancer. Lymphedema is a chronic swelling of the limb on the surgical side that may present immediately or many years after treatment [65,66]. More recent estimates suggest an incidence rate of around 20%, with higher rates found in studies with longer follow-up [67,68]. Lymphedema is a known consequence of surgical and radiotherapeutic techniques and is known to have deleterious effects on QoL [69]. Among systemic factors, obesity has been associated with increased lymphedema risk [70]. While PA has been traditionally viewed as a possible risk factor, PA has not been associated with lymphedema in prospective research and more recent evidence suggests a possible protective effect of PA [71].

Upper limb morbidity occurs frequently following treatment for breast cancer [71,72] and recent evidence suggests symptoms such as pain and shoulder dysfunction are more prevalent than lymphedema [72]. Although upper limb morbidity is reduced with newer techniques such as Sentinel Lymph Node Biopsy, studies have shown that a majority of breast cancer survivors have at least one upper limb symptom (e.g., numbness, pain, weakness, swelling, stiffness) in the long term [67].

Peripheral neuropathy is a condition that results from damage to or dysfunction of the peripheral nerves [73]. In breast cancer survivors this damage may occur from administration of a neurotoxic chemotherapeutic agent [73]. Sensory symptoms associated with chemotherapy induced peripheral neuropathy include numbness, tingling and pain that presents in the distal aspects of the upper and lower extremities, often described as a stocking/glove distribution [74]. Motor symptoms associated with the condition may include upper and lower limb weakness, impaired proprioception and balance. Functional impairments may result in difficulty performing fine motor tasks, walking and increase the risk falling [74]. Thus, breast cancer survivors experiencing treatment related effects such as lymphedema, upper limb morbidity and peripheral neuropathy, may have unique challenges that impact their PA, HRF, and PROs.

### Determinants of physical activity and health-related fitness

The aim of this study is to develop a comprehensive understanding of the determinants of PA in breast cancer survivors across the survivorship continuum. A social ecological approach and a theoretical framework [i.e., Theory of Planned Behavior (TPB)] will be used to identify key determinants of both the adoption and maintenance of PA across the continuum of breast cancer survivorship including social cognitive, demographic, personal, biological, medical, and environmental factors. The social ecological approach provides a broad framework to examine the multiple effects and interrelatedness of these factors at all levels of influence (i.e., individual, interpersonal, organizational, community, and society). Moreover, the TPB is one of the most widely tested theoretical frameworks within the PA and cancer literature.

More research is necessary to determine the specific relationship between these demographic variables and aspects of PA, including specifically the type, frequency, duration and intensity, as well as the timing of PA across the breast cancer continuum. Less is known about the role of medical factors as determinants, although PA participation consistently decreases with advanced breast cancer and during treatments. No research to date has specifically examined the role of biological factors as determinants of PA behavior during and after treatment. Therefore, examining biomarkers as PA determinants may provide a unique insight into the role of cancer-related biology as a determinant of PA.

Given the scarcity of literature on the myriad of determinants of PA for breast cancer survivors, the proposed prospective cohort study will generate new knowledge and be instrumental in formulating eventual clinical and community-based programming for breast cancer survivors. We will more clearly elucidate the complex interplay between a range of determinants and PA adoption and maintenance. Ultimately, the determinants project will enable us to achieve more effectively targeted interventions that help breast cancer survivors achieve healthy levels of PA and HRF.

In summary, the AMBER Study will establish a cohort in which we will conduct five initial studies that address the outcomes, determinants, mechanisms, and moderators of PA and HRF in breast cancer survivors. The AMBER Study will answer wide-ranging questions related to PA and HRF in breast cancer survivors. The result will be a unique data source containing data from objective and gold-standard measures that has not previously been created. This study will provide insight into a multitude of future research questions. Other important questions will arise for which the AMBER Study could provide timely answers. The ultimate goal of this research is to identify how PA and HRF can be used to inform clinical and public health recommendations for improving outcomes after breast cancer.

## Competing interests

The authors declare that they have no competing interests.

## Authors’ contributions

KSC and CMF conceived the study, developed the study methods, and drafted the manuscript. JKV was in charge of the patient-reported outcomes and edited the manuscript. SNC was responsible for developing the procedures regarding the determinants of physical activity and edited the manuscript. MLM was in charge of the lymphedema and upper body function assessments and edited the manuscript. GJB conceptualized and decided on the appropriate health-related fitness assessments and edited the manuscript. JRM was in charge of the disease outcomes and edited the manuscript. YYasui and YYuan were responsible for the statistical analyses and edited the manuscript. CEM provided expertise on the objective measures of physical activity and sedentary behavior and edited the manuscript, DCWL provided input on the biologic assays to be conducted on the biomarkers for this study, DC helped develop the study methods and edited and reviewed the manuscript. All authors read and approved the final manuscript.

## Pre-publication history

The pre-publication history for this paper can be accessed here:

http://www.biomedcentral.com/1471-2407/12/525/prepub
